# The Stockholm early detection of cancer study (STEADY-CAN): rationale, design, data collection, and baseline characteristics for 2.7 million participants

**DOI:** 10.1007/s10654-024-01192-8

**Published:** 2025-01-05

**Authors:** Elinor Nemlander, Eliya Abedi, Per Ljungman, Jan Hasselström, Axel C. Carlsson, Andreas Rosenblad

**Affiliations:** 1 Department of Neurobiology, Care Sciences and Society, Division of Family Medicine and Primary Care, Karolinska Institutet, Stockholm, Sweden; 2 Academic Primary Health Care Centre, Region Stockholm, Stockholm, Sweden; 3 Regional Cancer Centre Stockholm-Gotland, Region Stockholm, Stockholm, Sweden; 4 Department of Cellular Therapy and Allogeneic Stem Cell Transplantation, Karolinska Comprehensive Cancer Centre, Karolinska University Hospital, and Division of Haematology, Department of Medicine Huddinge,, Karolinska Institutet, Stockholm, Sweden; 5 Department of Statistics, Uppsala University, Uppsala, Sweden; 6 Department of Medical Sciences, Division of Clinical Diabetology and Metabolism, Uppsala University, Uppsala, Sweden

**Keywords:** Anaemia, Cancer, Cohort study, Primary care, Laboratory tests, Real world data

## Abstract

The Stockholm Early Detection of Cancer Study (STEADY-CAN) cohort was established to investigate strategies for early cancer detection in a population-based context within Stockholm County, the capital region of Sweden. Utilising real-world data to explore cancer-related healthcare patterns and outcomes, the cohort links extensive clinical and laboratory data from both inpatient and outpatient care in the region. The dataset includes demographic information, detailed diagnostic codes, laboratory results, prescribed medications, and healthcare utilisation data. Since its inception, STEADY-CAN has collected longitudinal data on 2,732,005 individuals aged ≥ 18 years old living in or having access to health care in Stockholm County during the years 2011–2021. Focusing on cancer, the cohort includes 140,042 (5.1%) individuals with incident cancer and a control group of 2,591,963 (94.9%) cancer-free individuals. The cohort’s diverse adult population enables robust analyses of early symptom detection, incidental findings, and the impact of comorbidities on cancer diagnoses. Utilizing the wide range of available laboratory data and clinical variables allow for advanced statistical analyses and adjustments for important confounding factors. The cohort’s primary focus is to improve understanding of the early diagnostic phase of cancer, offering a crucial resource for studying cancer detection in clinical practice. Its comprehensive data collection provides unique opportunities for research into comorbidities and cancer outcomes, making the cohort a useful resource for ongoing cancer surveillance and public health strategies. The present study gives a detailed description of the rationale for creating the STEADY-CAN cohort, its design, the data collection procedure, and baseline characteristics of collected data.

## Introduction

Cancer remains a leading cause of morbidity and mortality worldwide, imposing a substantial burden on healthcare systems [1]. Early cancer detection is crucial for improving patient outcomes, reducing treatment costs, and enhancing overall healthcare quality [2]. In this context, primary health care (PHC) plays a vital role, where the prompt recognition of cancer risk can significantly influence the patient’s prognosis [3, 4].

Improving the process of cancer diagnosis in PHC is a complex challenge that requires further research. General practitioners (GPs) have to handle the delicate balance to be struck between the need for timely and accurate diagnosis for patients with serious illnesses, and the potential harm caused by unnecessary diagnostic tests in patients with low-risk [5]. Additionally, cancer stands out as one of the most frequently missed or delayed diagnoses [6, 7], causing harm to individuals and clouding the reputation of PHC providers.

Knowledge of the epidemiology of symptoms and signs associated with cancer diagnosis in PHC has progressed over the past decade, primarily with substantial contributions from studies in the UK [8–10]. These studies, which are partly or wholly based on Reed codes, underscore the necessity for risk prediction tools using International Classification of Diseases and Related Health Problems (ICD) codes that are more widely used. Furthermore, there is a need for more objective measures beyond diagnostic codes to identify cancer with a higher accuracy [11, 12].Clinical decision-making in PHC relies predominantly on risk assessment [13]. Symptoms traditionally seen as warning signs for cancer, such as rectal bleeding and dysphagia, are common in the general population [14, 15], also in patients without cancer. The challenge intensifies as many patients subsequently diagnosed with cancer often present with non-specific symptoms rather than those conventionally deemed alarming or *red-flag* [16]*.* Individuals with non-specific cancer symptoms often have a poorer prognosis and are diagnosed with cancer at a more advanced stage than those exhibiting *red-flag* symptoms [17, 18]. Increased frequency of medical visits and anaemia are two prevalent non-specific cancer indicators [19]. However, both anaemia and increased frequency of medical visits are common features among patients seeking PHC without cancer [20].

In an earlier study, we showed that frequency of visits in addition to registered diagnoses can predict colorectal cancer [21], and believe that combinations of laboratory values, visit frequencies, and registered diagnoses in primary care may be even more successful in detecting different types of cancer. Objective markers could facilitate the practical application of risk prediction tools in PHC. GPs have access to extensive electronic medical record data, healthcare contacts, and routinely collected laboratory data for a large part of the population, potentially holding valuable information. Assessments of haemoglobin are among the most common routine laboratory analyses and are performed on broad indications in clinical practice. This includes both emergency settings and regular monitoring of patients with various diseases and symptoms. The extent of haemoglobin testing in healthcare is unknown but can provide crucial information. Therefore, we decided to investigate the patterns and implications of haemoglobin testing in healthcare to better understand its potential in guiding clinical decisions.

In 2022, Sweden had a population of about 10.5 million, of which nearly 25% (2.4 million) resided in the Stockholm Region. The healthcare system in Sweden operates with public funding, ensuring universal access to medical services for all residents. The administration of healthcare services in Sweden is primarily decentralized to the regional level, with 21 regions overseeing the delivery of healthcare across the country. Healthcare providers have an obligation to document diagnoses and report information related to healthcare utilization, reasons for hospitalizations, and visits to primary and specialist care. This includes the use of diagnostic codes, prescription information, and socio-demographic data. Sweden possesses a high-quality cancer registry since more than 65 years, ensuring completeness and reliability in cancer registrations [22].

The Stockholm Early Detection of Cancer Study (STEADY-CAN) is a project within Stockholm County aimed at identifying symptoms and signs in PHC that can improve opportunities for early cancer detection. This study provides an overview of the goals and objectives of the STEADY-CAN cohort, details about the extraction of medical record data, laboratory data, and linked data sources. Several studies aiming to develop risk assessment tools for different types of cancer with the STEADY-CAN data are planned. Additionally, we provide an assessment of regional coverage and representativeness.

### Overarching objectives of STEADY-CAN

The overarching objectives of STEADY-CAN revolve around enhancing the research and evidence base concerning cancer risk assessment in PHC using real world data. The initiative aims to aid GPs in early cancer detection focusing on crucial research methodologies, including clinical epidemiology, artificial intelligence, machine learning, diagnostic tests, risk assessment tool development, and health services research.

The initial specific goals include:

i. Establishing the prevalence of incident anaemia among adult patients visiting PHC in the Stockholm region and evaluating its predictive ability for overall and specific forms of cancer. These analyses consider modifying factors such as sex, age, comorbidity, and other routinely collected information.
ii. Investigate whether an increasing frequency of medical visits is an independent indicator of undiagnosed cancer in PHC, or if it serves as a risk marker for undiagnosed cancer when associated with comorbidities and other factors.
iii. Evaluating the predictive potential for cancer of a standardised set of laboratory tests, routinely conducted in PHC settings as part of cancer investigations, using machine learning techniques.

## Methods

### Construction and content

To address our research questions, a population-based cohort, the Stockholm Early Detection of Cancer Study (STEADY-CAN) cohort, of individuals aged ≥ 18 years old residing or having access to healthcare in Stockholm County has been established. The data extraction period spanned from January 1, 2011, to December 31, 2021. For those turning 18 years old during the study period, data were only included from the date when they turned 18.

An outcome of main interest was incident cancer during the 10-years period from January 1, 2012 to December 31, 2021, defined as having no previous cancer during the outcome period and back to January 1, 1992.

Data were obtained from various prospectively collected data sources and linked using the participants’ unique Swedish personal identification numbers (PINs) [23] to enable large-scale population-based analyses. Cancer data were obtained from the Swedish Cancer Registry (SCR) [22], while data on diagnoses and symptom codes from PHC, specialized outpatient and inpatient care, socio-economic data based on MOSAIC areas [24, 25], and visitor statistics were collected from Region Stockholm’s healthcare administration's databases (VAL) [26]. Additionally, data on dispensed medications relevant to anaemia were collected from the National Prescribed Drug Register [27]. Finally, cancer-related laboratory data were obtained from the active clinical laboratories in the Stockholm Region (Karolinska University Laboratory, Unilabs, and SYNLAB Medilab).

**Fig. 1:**
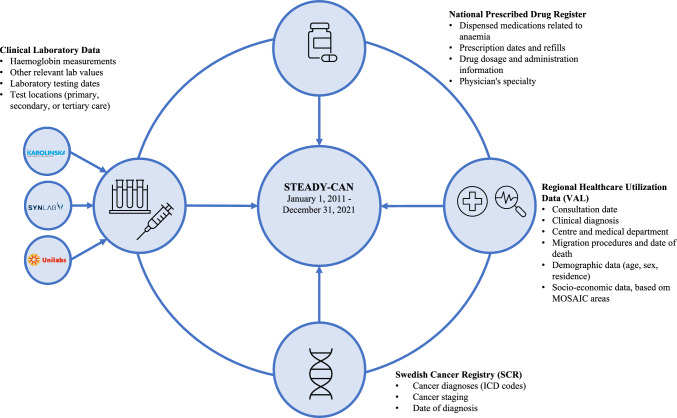
Data sources and cohort construction in the Stockholm early detection of cancer study (STEADY-CAN)

All data were submitted to and linked by the Swedish National Board of Health and Welfare. Once the requested data had been linked, it was pseudonymized by replacing the PINs with randomly generated serial numbers to ensure patient privacy and confidentiality. The key for linking the serial numbers to the PINs was stored by the National Board of Health and Welfare, allowing for potential modifications, follow-ups, and/or new linkages and updates to the STEADY-CAN database. An overview of the data collection procedure is given in Fig. 1.

The entire STEADY-CAN project data set was transferred to the responsible researchers after pseudonymization. Data were stored on encrypted and secure servers within the Stockholm Region. Data analyses adhered to university regulations, and the project was reviewed and approved by the Swedish Ethical Review Authority (Dnr. 2021-05069 and 2023-00704-02).

### Management of healthcare utilization data

Data on healthcare interactions in Stockholm County are automatically compiled and stored in the region’s healthcare administration’s databases–VAL [26]. The intended uses of VAL include tasks as healthcare planning, determining compensation for healthcare providers, and evaluating the quality of care. All residents in Region Stockholm with documented healthcare interactions are registered in VAL, which also contains information on dates of death and migrations in and out of the region. Both public and private healthcare providers within the region report to VAL.

Data on each healthcare visit was accompanied by the date of the visit (and, if applicable, discharge date), the visited unit/medical department, and established diagnoses according to the ICD 10th revision coding system (ICD-10). All ICD-10 codes were retrieved for each individual from January 1, 2011, to December 31, 2021. Additionally, ICD 10-codes for anaemia diagnoses (D50.x − D64.x and Y44.x) were retrieved for the period January 1996 (or from the earliest possible date) until December 31, 2021, to allow for exclusion of previously known anaemia or hemoglobinopathy. To take account of comorbidity, all data needed to calculate the Charlson Comorbidity Index (CCI) [28] were collected. In addition, comprehensive data on chronic disorders and diagnostic codes from nearly all healthcare contacts were collected, allowing for adjustments for relevant comorbidities based on the specific study designs. Demographic data included sex and date of birth, migration in and out of the region, and which PHC centre an individual was registered with on December 31 each year during the window of extraction. Additionally, socio-economic data based on MOSAIC areas [24] were obtained per individual at each healthcare event. For privacy reasons, the date of birth obtained by the researchers after pseudonymization was truncated to only show year and month of birth, and the date of birth used in the analyses was then set to the 15th of each month.

**Table 1:** Demographic and clinical characteristics of the STEADY-CAN cohort

Variable	Cases		Controls	
	Male	Female	Male	Female
	n = 73,711 (52.6%)	n = 66,331 (47.4%)	n = 1,256,572 (48.5%)	n = 1,335,391 (51.5%)
Age (years) at date of inclusion,^a^ mean (SD)	61.2 (13.0)	59.0 (15.5)	39.4 (17.9)	40.5 (19.7)
18–44 years old, n (%)	7656 (10.4)	12,939 (19.5)	811,593 (64.6)	843,571 (63.2)
45–64 years old, n (%)	34,976 (47.5)	27,222 (41.0)	316,944 (25.2)	309,934 (23.2)
65–79 years old, n (%)	27,439 (37.2)	21,341 (32.2)	99,098 (7.9)	122,682 (9.2)
≥ 80 years old, n (%)	3640 (4.9)	4829 (7.3)	28,937 (2.3)	59,204 (4.4)
Moving into Stockholm County during 2012–2021, n (%)	4449 (6.0)	4353 (6.6)	235,545 (18.7)	254,708 (19.1)
Moving in permanently, n (%)	3067 (4.2)	3004 (4.5)	137,777 (11.0)	151,429 (11.3)
Moving out of Stockholm County during 2012–2021, n (%)	3734 (5.1)	3381 (5.1)	165,283 (13.2)	176,007 (13.2)
Moving out permanently, n (%)	3056 (4.1)	2790 (4.2)	121,298 (9.7)	133,363 (10.0)
Deceased in Stockholm County during 2012–2021, n (%)	15,245 (20.7)	13,803 (20.8)	47,094 (3.7)	54,552 (4.1)
Total length of inpatient visits during 2011–2021, mean (SD)	17.8 (41.6)	19.5 (40.0)	4.3 (31.2)	5.1 (26.5)
Anaemia diagnosis during 1996–2011, n (%)^b^	1066 (1.4)	1526 (2.3)	14,478 (1.2)	29,370 (2.2)
CCI (points) excl. cancer diagnoses 2011–2021, mean (SD)^c^	1.01 (1.69)	0.76 (1.37)	0.37 (1.05)	0.34 (0.93)
0 points, n (%)	43,584 (59.1)	42,171 (63.6)	1,025,803 (81.6)	1,083,252 (81.1)
1–2 points, n (%)	18,839 (25.6)	17,380 (26.2)	168,424 (13.4)	199,588 (14.9)
3–4 points, n (%)	7209 (9.8)	4735 (7.1)	42,551 (3.4)	37,720 (2.8)
≥ 5 points, n (%)	4079 (5.5)	2045 (3.1)	19,794 (1.6)	14,831 (1.1)

CCI, Charlson Comorbidity Index; PHC, primary health care; ICD-10, International Statistical Classification of Diseases and Related Health Problems, 10th Revision; SD, standard deviation. ^a^ January 1, 2012 or, if aged < 18 years old at this date, the first day of the month after the individual turned 18 years old. ^b^ ICD-10 codes D50-D64, Y44. ^c^ CCI was constructed using ICD-10 coding according to Quan et al. and weights from Charlson et al., excluding the comorbidities “Any malignancy, including lymphoma and leukaemia, except malignant neoplasm of skin” and “Metastatic solid tumor”

### Management of prescribed medications

Data on medications relevant to anaemia were included in the STEADY-CAN cohort using their generic names and active substance ATC codes, as outlined in Table 1, along with the quantity of dispensed medication defined daily doses (DDD) [29] and dispensing dates. This information was obtained from the National Prescribed Drug Register [27], a nationwide registry compiling data on all prescribed medications dispensed by Swedish pharmacies. The register had an almost complete coverage (> 99.7%) for all dispensed medications but did not include information on over-the-counter medications or medications for outpatient or hospital care administered within healthcare facilities (Table 2). Data on medication dispensing were collected for each individual from January 1, 2011, to December 31, 2021.

**Table 2:** Overview of selected ATC codes, their corresponding drug classes, and descriptions

ATC Code	Name	Description
M01A	Anti-inflammatory and antirheumatic products, non-steroids	Medications used for inflammation and rheumatic conditions
N02BA01	Acetylsalicylic acid	Used for pain relief and reducing inflammation
N06AB	Selective serotonin reuptake inhibitors (SSRIs)	Antidepressants that increase serotonin levels in the brain
B03	Anti-anaemic preparations	Medications used to treat or prevent anaemia
B02	Haemostatics	Drugs that help control bleeding
B01	Antithrombotic agents	Medications that reduce the formation of blood clots

### Laboratory data extraction

The second key component of the STEADY-CAN project focuses on the integration of laboratory data. Three main laboratories (SYNLAB Sverige AB part of the SYNLAB group, Unilabs AB, and Karolinska University Laboratory) were responsible for performing nearly all clinical laboratory tests in the region throughout the study period. Each laboratory provider retrieved the specified laboratory tests during the study period encompassing the entire region. The main interest of the present study was haemoglobin measurements in primary care, secondary care, or tertiary care during 2011–2021. All haemoglobin test results during this period, along with additional laboratory measurements routinely associated with cancer investigation, were included in the analyses, guiding continuous patient assessment, and identifying relevant outcomes, as outlined in Table 5. Each laboratory test, aside from test specifics, included a unique patient identifier [23], test date, ordering unit, and analysis method. Only venous haemoglobin measurements were considered. Both inter- and intra-laboratory variabilities were considered negligible, with routine quality checks and standardization assessments of the labs regularly performed by the national external quality assessment (EQA) provider Equalis AB (www.equalis.se).

### Management of the Swedish Cancer Registry

The SCR, established in 1958, is one of the oldest disease registers in the world and has high validity [22]. All physicians, including pathology laboratories, in Sweden are obliged by law to report all incident cases of cancer to the SCR. The registry covers all types of cancer, except non-melanocytic skin cancer (ICD10 code C44) and includes a wide range of variables. For the STEADY-CAN cohort, data on all cancer diagnoses with a diagnosis date during the period from January 1, 1992 to December 31, 2021 were collected. Besides date of diagnosis, data were obtained on cancer stages at diagnosis according to the TNM (Tumour, Node, Metastasis) Classification of Malignant Tumours and date of death.

## Results

**Fig. 2:**
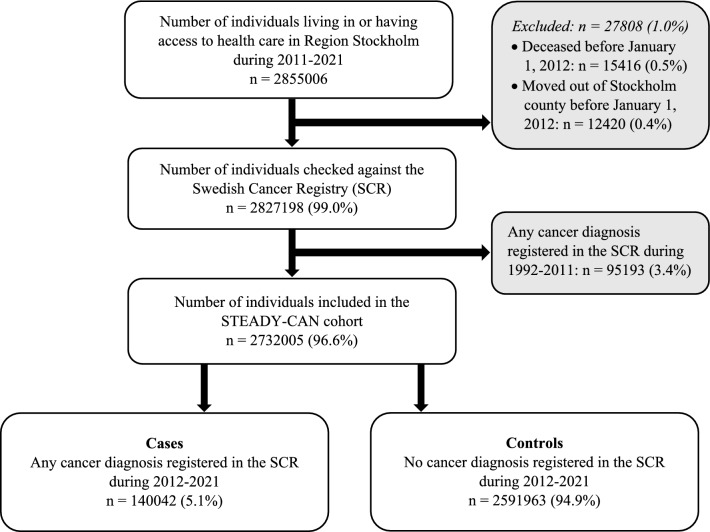
Flowchart of the inclusion process for the STEADY-CAN cohort

The STEADY-CAN cohort identified a total of 2,855,006 adult individuals living in or having access to health care in Region Stockholm during 2011–2021. After excluding individuals who had died or moved out of Stockholm County before January 1, 2012 (n = 27,808), and those registered with any cancer diagnosis (except non-melanocytic skin cancer) in the SCR during 1992–2011 (n = 95,193), a total of 2,732,005 individuals were included in the STEADY-CAN cohort (Fig. 2).

In total, 140,042 (5.1%) patients with incident cancer, 73,711 (52.6%) men and 66,331 (47.4%) women, were identified during the outcome period 2012–2021. All incident organ specific cases of cancer are given in Table 3. The most common cancer diagnoses among men were prostate cancer (39.0%), colorectal cancer (10.2%), and melanoma (6.2%). Among women, the most common diagnoses were breast cancer (31.7%), colorectal cancer (10.3%), and lung cancer (7.6%). Cancer-free controls comprised all individuals who did not receive a cancer diagnosis during the study period and had no previous cancer registered in the SCR during 1992–2011, totalling 2,591,963 (94.9%) individuals.

**Table 3:** Incident organ specific cases of cancer (ICD 10 codes C00-C43, C45-C97, D45-D47) among cases 2012–2021, n (%)

Type of cancer	ICD-10 code	Total	Male	Female
		n = 140,042 (100%)	n = 73,711 (52.6%)	n = 66,331 (47.4%)
Malignant neoplasms of lip, oral cavity and pharynx	C00-C14	2871 (2.1)	1746 (2.4)	1125 (1.7)
Lip and oral cavity^a^	C00-C06	1672 (1.2)	954 (1.3)	718 (1.1)
Malignant neoplasms of digestive organs	C15-C26	25,580 (18.3)	13,892 (18.8)	11,688 (17.6)
Malignant neoplasm of oesophagus^a^	C15	1121 (0.8)	809 (1.1)	312 (0.5)
Malignant neoplasm of stomach^a^	C16	1879 (1.3)	1152 (1.6)	727 (1.1)
Colorectal cancer	C18-C20	14,122 (10.1)	7409 (10.1)	6713 (10.1)
Malignant neoplasm of colon or rectosigmoid junction^a,b^	C18-C19	9531 (6.8)	4660 (6.3)	4871 (7.3)
Malignant neoplasm of rectum^a^	C20	4663 (3.3)	2791 (3.8)	1872 (2.8)
Malignant neoplasm of liver and intrahepatic bile ducts^a^	C22	2307 (1.6)	1628 (2.2)	679 (1.0)
Malignant neoplasm of pancreas^a^	C25	3662 (2.6)	1805 (2.4)	1857 (2.8)
Malignant neoplasms of respiratory and intrathoracic organs	C30-C39	10,015 (7.2)	4805 (6.5)	5210 (7.9)
Malignant neoplasm of bronchus and lung^a^	C34	9039 (6.5)	4100 (5.6)	4939 (7.4)
Malignant neoplasms of bone and articular cartilage	C40-C41	292 (0.2)	164 (0.2)	128 (0.2)
Malignant melanoma of skin^a^	C43	8857 (6.3)	4500 (6.1)	4357 (6.6)
Malignant neoplasms of mesothelial and soft tissue	C45-C49	1195 (0.9)	642 (0.9)	553 (0.8)
Malignant neoplasm of breast^a^	C50	20,895 (14.9)	121 (0.2)	20,774 (31.3)
Malignant neoplasms of female genital organs	C51-C58	7607 (5.4)	N/A	7607 (11.5)
Gynaecologic cancer	C51–C54, C559, C569, C570	7522 (5.4)	N/A	7522 (11.3)
Malignant neoplasm of cervix uteri^a^	C53	1725 (1.2)	N/A	1725 (2.6)
Malignant neoplasm of corpus uteri^a^	C54	3234 (2.3)	N/A	3234 (4.9)
Malignant neoplasm of ovary^a^	C56	1305 (0.9)	N/A	1305 (2.0)
Malignant neoplasms of male genital organs	C60-C63	30,019 (21.4)	30,019 (40.7)	N/A
Malignant neoplasm of prostate^a^	C61	28,380 (20.3)	28,380 (38.5)	N/A
Malignant neoplasm of testis	C62	1391 (1.0)	1391 (1.9)	N/A
Malignant neoplasms of urinary tract	C64-C68	9384 (6.7)	6588 (8.9)	2796 (4.2)
Malignant neoplasm of kidney, except renal pelvis^a^	C64	2935 (2.1)	1929 (2.6)	1006 (1.5)
Malignant neoplasm of bladder^a^	C67	5894 (4.2)	4330 (5.9)	1564 (2.4)
Malignant neoplasms of eye, brain and other parts of central nervous system	C69-C72	5335 (3.8)	2560 (3.5)	2775 (4.2)
Brain and nervous system^a^	C70-C72	4260 (3.0)	1999 (2.7)	2261 (3.4)
Malignant neoplasm of meninges	C70	1480 (1.1)	436 (0.6)	1044 (1.6)
Malignant neoplasm of brain	C71	2147 (1.5)	1250 (1.7)	897 (1.4)
Malignant neoplasms of thyroid and other endocrine glands	C73-C75	4548 (3.2)	1412 (1.9)	3136 (4.7)
Malignant neoplasm of thyroid gland^a^	C73	1587 (1.1)	384 (0.5)	1203 (1.8)
Malignant neoplasm of other endocrine glands and related structures	C75	2895 (2.1)	1001 (1.4)	1894 (2.9)
Malignant neoplasms of ill-defined, secondary and unspecified sites	C76-C80	1988 (1.4)	756 (1.0)	1232 (1.9)
Malignant neoplasms, stated or presumed to be primary, of lymphoid, haematopoietic and related tissue	C81-C96	9691 (6.9)	5590 (7.6)	4101 (6.2)
Non-Hodgkin lymphoma^a^	C82-C85	4318 (3.1)	2469 (3.3)	1849 (2.8)
Non-follicular lymphoma	C83	2494 (1.8)	1479 (2.0)	1015 (1.5)
Multiple myeloma and malignant plasma cell neoplasms	C90	1721 (1.2)	1012 (1.4)	709 (1.1)
Leukaemia^a^	C91-C95	2815 (2.0)	1666 (2.3)	1149 (1.7)
Lymphoid leukaemia	C91	1615 (1.2)	1009 (1.4)	606 (0.9)
Myelodysplastic syndromes and Other neoplasms of uncertain or unknown behaviour of lymphoid, haematopoietic and related tissue	D46-D47	1873 (1.3)	984 (1.3)	889 (1.3)

ICD-10, International Statistical Classification of Diseases and Related Health Problems, 10th Revision; N/A, not applicable. Includes only individuals without any previous cancer registered in the Swedish Cancer Register during the period from January 1st, 1992 to the date of diagnosis of the specified cancer. No one had the diagnoses “Malignant neoplasms of independent (primary) multiple sites” (ICD-10 code C97) or “Polycythaemia vera” (ICD-10 code D45). ^a^Included in the GLOBOCAN 2020 list with ≥ 1.0% of all new cancer cases worldwide during 2020. ^b^In total, n = 3 males and n = 1 female were diagnosed with “Malignant neoplasm of rectosigmoid junction” (ICD-10 code C19)

The mean age in the cohort was higher among cases (men: 61.2 years, women: 59.0 years) compared to controls (men: 39.4 years, women: 40.5 years). A higher proportion of cases died between 2012 and 2021, 20.7% of men and 20.8% of women compared to 3.7% and 4.1% of men and women controls, respectively. Cases also had a lower proportion of individuals who had moved into or out of Stockholm compared to controls.

The CCI score, excluding cancer diagnoses from 2011 to 2021, was higher among cases (men: 1.01 points, women: 0.76 points) than controls (men: 0.37 points, women: 0.34 points), indicating a higher burden of comorbidities among cases. A higher proportion of cases had three or more CCI points (men: 15.3%, women: 10.2%) compared to controls (men: 5.0%, women: 3.9%).

Table 4 presents the diagnoses registered for individuals in the STEADY-CAN cohort during inpatient or outpatient visits between 2011 and 2021 for both cases and controls. As the controls are unmatched to cases and there is a significant age difference between groups, the differences between cases and controls are not of primary analytical interest in this context. We include all available controls to demonstrate the full scope of data within the cohort, facilitating future matching and comparisons. How these data will be used in specific analyses is a matter for future analyses, and it is important to emphasize that this table forms part of the initial description of the cohort’s structure and content.

**Table 4:** Diagnoses registered for individuals in the STEADY-CAN cohort at in- or outpatient visits during 2011–2021

Diagnosis	ICD 10 code	Cases		Controls	
		Male	Female	Male	Female
		n = 73711 (52.6%)	n = 66331 (47.4%)	n = 1256572 (48.5%)	n = 1335391 (51.5%)
Certain infectious and parasitic diseases	A00-B99	24929 (33.8)	25078 (37.8)	307482 (24.5)	413635 (31.0)
AIDS/HIV^a^	B20–B22, B24	112 (0.2)	36 (0.1)	882 (0.1)	696 (0.1)
Other viral diseases^b^	B25-B34	4227 (5.7)	5579 (8.4)	81101 (6.5)	123319 (9.2)
In situ neoplasms	D00-D09	1954 (2.7)	6167 (9.3)	4120 (0.3)	22740 (1.7)
Benign neoplasms	D10-D36	14986 (20.3)	18908 (28.5)	131922 (10.5)	243081 (18.2)
Diseases of the blood and blood-forming organs and certain disorders involving the immune mechanism	D50-D89	15066 (20.4)	14605 (22.0)	52816 (4.2)	108072 (8.1)
Nutritional anaemias	D50-D53	3841 (5.2)	4391 (6.6)	16070 (1.3)	54115 (4.1)
Haemolytic anaemias	D55-D59	171 (0.2)	196 (0.3)	1559 (0.1)	3310 (0.2)
Aplastic and other anaemias	D60-D64	10983 (14.9)	9604 (14.5)	29527 (2.3)	49164 (3.7)
Endocrine, nutritional and metabolic diseases	E00-E90	26367 (35.8)	26860 (40.5)	194369 (15.5)	320681 (24.0)
Disorders of thyroid gland^b^	E00-E07	2534 (3.4)	8914 (13.4)	20867 (1.7)	116531 (8.7)
Diabetes mellitus^b^	E10-E14	10982 (14.9)	6751 (10.2)	77180 (6.1)	57286 (4.3)
Diabetes without chronic complication^a^	E10.0, E10.1, E10.6, E10.8, E10.9, E11.0, E11.1, E11.6, E11.8, E11.9, E12.0, E12.1, E12.6, E12.8, E12.9, E13.0, E13.1, E13.6, E13.8, E13.9, E14.0, E14.1, E14.6, E14.8, E14.9	10875 (14.8)	6704 (10.1)	75884 (6.0)	56521 (4.2)
Diabetes with chronic complication^a^	E10.2–E10.5, E10.7, E11.2–E11.5, E11.7, E12.2–E12.5, E12.7, E13.2–E13.5, E13.7, E14.2–E14.5, E14.7	3841 (5.2)	2022 (3.0)	28090 (2.2)	18012 (1.3)
Metabolic disorders^b^	E70-E90	16216 (22.0)	14148 (21.3)	98993 (7.9)	106652 (8.0)
Mental and behavioural disorders	F00-F99	16216 (22.0)	20592 (31.0)	302274 (24.1)	444954 (33.3)
Dementia^a^	F00–F03, F05.1, G30, G31.1	1937 (2.6)	1921 (2.9)	15787 (1.3)	27129 (2.0)
Mood [affective] disorders^b^	F30-F39	5092 (6.9)	8352 (12.6)	101646 (8.1)	177147 (13.3)
Neurotic, stress-related and somatoform disorders^b^	F40-F48	6798 (9.2)	13403 (20.2)	170795 (13.6)	332556 (24.9)
Diseases of the nervous system	G00-G99	17435 (23.7)	18775 (28.3)	190895 (15.2)	292103 (21.9)
Hemiplegia or paraplegia^a^	G04.1, G11.4, G80.1, G80.2, G81-G82, G83.0–G83.4, G83.9	1237 (1.7)	884 (1.3)	8044 (0.6)	7011 (0.5)
Diseases of the eye and adnexa	H00-H59	27476 (37.3)	28578 (43.1)	257194 (20.5)	357267 (26.8)
Disorders of conjunctiva^b^	H10-H13	5076 (6.9)	6179 (9.3)	68120 (5.4)	103303 (7.7)
Diseases of the ear and mastoid process	H60-H95	22210 (30.19	20346 (30.7)	236749 (18.8)	292902 (21.9)
Diseases of external ear^b^	H60-H62	12065 (16.4)	10629 (16.0)	132676 (10.6)	149379 (11.2)
Diseases of middle ear and mastoid^b^	H65-H75	3079 (4.2)	3699 (5.6)	54494 (4.3)	78380 (5.9)
Diseases of the circulatory system	I00-I99	39683 (53.8)	33482 (50.5)	281282 (22.4)	307716 (23.0)
Hypertensive diseases^b^	I10-I15	30140 (40.9)	24263 (36.6)	195693 (15.6)	204477 (15.3)
Myocardial infarction^a^	I21-I22, I25.2	5681 (7.7)	2151 (3.2)	31409 (2.5)	17269 (1.3)
Congestive heart failure^a^	I09.9, I11.0, I13.0, I13.2, I25.5, I42.0, I42.5–I42.9, I43, I50, P29.0	6426 (8.7)	4613 (7.0)	34813 (2.8)	33752 (2.5)
Cerebrovascular disease^a^	G45-G46, H34.0, I60–I69	6528 (8.9)	4753 (7.2)	38054 (3.0)	38455 (2.9)
Peripheral vascular disease^a^	I70-I71, I73.1, I73.8, I73.9, I77.1, I79.0, I79.2, K55.1, K55.8, K55.9, Z95.8, Z95.9	3840 (5.2)	2113 (3.2)	18346 (1.5)	13663 (1.0)
Diseases of the respiratory system	J00-J99	30350 (41.2)	31524 (47.5)	438770 (34.9)	561527 (42.0)
Acute upper respiratory infections^b^	J00-J06	14165 (19.2)	18204 (27.4)	278582 (22.2)	409825 (30.7)
Other acute lower respiratory infections^b^	J20-J22	6120 (8.3)	7304 (11.0)	67511 (5.4)	102794 (7.7)
Chronic pulmonary disease^a^	I27.8, I27.9, J40–J47, J60–J67, J68.4, J70.1, J70.3	7995 (10.8)	9366 (14.1)	79190 (6.3)	117630 (8.8)
Chronic lower respiratory diseases^b^	J40-J47	7892 (10.7)	9271 (14.0)	78567 (6.3)	116960 (8.8)
Diseases of the digestive system	K00-K93	30000 (40.7)	27251 (41.1)	275322 (21.9)	348353 (26.1)
Peptic ulcer disease^a^	K25–K28	2231 (3.0)	1526 (2.3)	10194 (0.8)	9461(0.7)
Mild liver disease^a^	B18, K70.0–K70.3, K70.9,K71.3–K71.5, K71.7, K73-K74, K76.0, K76.2–K76.4, K76.8, K76.9, Z94.4	2375 (3.2)	1469 (2.2)	17331 (1.4)	13968 (1.0)
Moderate or severe liver disease^a^	I85.0, I85.9, I86.4, I98.2, K70.4, K71.1, K72.1, K72.9, K76.5, K76.6, K76.7	953 (1.3)	464 (0.7)	2972 (0.2)	1850 (0.1)
Diseases of the skin and subcutaneous tissue	L00-L99	29667 (40.2)	30386 (45.8)	378707 (30.1)	499475 (37.4)
Infections of the skin and subcutaneous tissue^b^	L00-L08	9322 (12.6)	9641 (14.5)	115961 (9.2)	124682 (9.3)
Dermatitis and eczema^b^	L20-L30	10733 (14.6)	11916 (18.0)	140920 (11.2)	207972 (15.6)
Diseases of the musculoskeletal system and connective tissue	M00-M99	39091 (53.0)	39262 (59.2)	553827 (44.1)	656453 (49.2)
Rheumatic disease^a^	M05-M06, M31.5, M32–M34, M35.1, M35.3, M36.0	1824 (2.5)	2433 (3.7)	9504 (0.8)	23977 (1.8)
Other joint disorders^b^	M20-M25	16022 (21.7)	18663 (28.1)	222041 (17.7)	294035 (22.0)
Other dorsopathies^b^	M50-M54	17295 (23.5)	19210 (29.0)	234041 (18.6)	315879 (23.7)
Other soft tissue disorders^b^	M70-M79	25837 (35.1)	27529 (41.5)	350637 (27.9)	443777 (33.2)
Diseases of the genitourinary system	N00-N99	30568 (41.5)	36050 (54.3)	245705 (19.6)	637102 (47.7)
Renal disease^a^	I12.0, I13.1, N03.2–N03.7, N05.2–N05.7, N18-N19, N25.0, Z49.0–Z49.2, Z94.0, Z99.2	5689 (7.7)	3130 (4.7)	26963 (2.1)	22396 (1.7)
Other diseases of urinary system^b^	N30-N39	14323 (19.4)	20350 (30.7)	74015 (5.9)	297992 (22.3)
Pregnancy, childbirth and the puerperium	O00-O99	N/A	3362 (5.1)	N/A	272633 (20.4)
Certain conditions originating in the perinatal period	P00-P96	95 (0.1)	106 (0.2)	610 (0.0)	1257 (0.1)
Congenital malformations, deformations and chromosomal abnormalities	Q00-Q99	1246 (1.7)	1481 (2.2)	20436 (1.6)	28730 (2.2)
Symptoms, signs and abnormal clinical and laboratory findings, not elsewhere classified	R00-R99	50092 (68.0)	46683 (70.4)	654755 (52.1)	810765 (60.7)
Symptoms and signs involving the circulatory and respiratory systems^b^	R00-R09	26410 (35.8)	26326 (39.7)	313685 (25.0)	380140 (28.5)
Symptoms and signs involving the digestive system and abdomen^b^	R10-R19	21109 (28.6)	24747 (37.3)	203281 (16.2)	368380 (27.6)
Symptoms and signs involving the skin and subcutaneous tissue^b^	R20-R23	19164 (26.0)	21238 (32.0)	212352 (16.9)	308976 (23.1)
General symptoms and signs^b^	R50-R69	29702 (40.3)	31503 (47.5)	313372 (24.9)	467304 (35.0)
Injury, poisoning and certain other consequences of external causes	S00-T98	30237 (41.0)	30161 (45.5)	456887 (36.4)	457516 (34.3)
Injuries involving multiple body regions^b^	T00-T07	826 (1.1)	813 (1.2)	12970 (1.0)	12520 (0.9)
Other and unspecified effects of external causes^b^	T66-T78	1524 (2.1)	2006 (3.0)	33690 (2.7)	47875 (3.6)
External causes of morbidity and mortality	V01-Y98	26428 (35.9)	27596 (41.6)	432089 (34.4)	426769 (32.0)
Factors influencing health status and contact with health services	Z00-Z99	54555 (74.0)	51246 (77.3)	703254 (56.0)	924908 (69.3)
Codes for special purposes	U00-U85	9883 (13.4)	8952 (13.5)	93512 (7.4)	118133 (8.8)

AIDS, acquired immunodeficiency syndrome; COPD, chronic obstructive pulmonary disease; HIV, human immunodeficiency virus; ICD-10, International Statistical Classification of Diseases and Related Health Problems, 10th Revision; N/A, not applicable. ^a^Included in the Charlson Comorbidity Index. ^b^Subchapter (block) with at least one disease included in the list of most common groups of diagnoses in primary health care in Stockholm during 2011

Table 5 shows the number of individuals in the STEADY-CAN cohort who underwent routine laboratory testing and the mean number of tests (with standard deviation) collected during the years 2011–2021, stratified by cases, controls, and sex. The test of main interest was B-Haemoglobin, where a total of 75% (n = 2,050,716) of individuals in STEADY-CAN had at least one venous haemoglobin test during the study period.

**Table 5:** Number of individuals included in the STEADY-CAN cohort having routine laboratory tests collected, and mean (SD) number of tests collected, during the years 2011–2021

Variable	Cases				Controls			
	Male		Female		Male		Female	
	n = 73,711 (52.6%)		n = 66,331 (47.4%)		n = 1,256,572 (48.5%)		n = 1,335,391 (51.5%)	
	n (%)	mean (SD)	n (%)	mean (SD)	n (%)	mean (SD)	n (%)	mean (SD)
B-EVF	61,143 (82.9)	16.9 (26.0)	55,213 (83.2)	16.8 (23.9)	841,569 (67.0)	4.7 (10.9)	978,043 (73.2)	5.8 (11.1)
B-Erythrocytes	60,924 (82.7)	16.6 (25.7)	55,039 (83.0)	16.5 (23.7)	840,025 (66.9)	4.6 (10.7)	975,518 (73.1)	5.8 (10.9)
B-Haemoglobin	64,368 (87.3)	24.4 (35.2)	57,326 (86.4)	24.2 (32.2)	903,351 (71.9)	5.8 (13.3)	1,025,671 (76.8)	7.0 (13.3)
B-Leukocytes	62,389 (84.6)	22.0 (33.7)	56,014 (84.4)	22.6 (31.1)	842,190 (67.0)	5.1 (12.4)	977,639 (73.2)	6.3 (12.5)
B-MCH	60,898 (82.6)	16.5 (25.7)	55,034 (83.0)	16.5 (23.7)	839,282 (66.8)	4.6 (10.7)	975,162 (73.0)	5.8 (10.9)
B-MCV	60,910 (82.6)	16.6 (25.7)	55,046 (83.0)	16.5 (23.7)	839,740 (66.8)	4.6 (10.7)	975,659 (73.1)	5.8 (10.9)
B-Platelets	62,172 (84.3)	20.7 (32.5)	55,801 (84.1)	21.0 (30.0)	849,336 (67.6)	4.9 (11.8)	984,188 (73.7)	6.1 (11.8)
B-Reticulocytes	13,442 (18.2)	0.50 (2.49)	11,178 (16.9)	0.43 (2.49)	59,004 (4.7)	0.08 (0.94)	101,614 (7.6)	0.13 (0.89)
P/S-Iron levels	27,540 (37.4)	1.13 (2.58)	27,882 (42.0)	1.26 (2.65)	277,713 (22.1)	0.50 (1.63)	468,929 (35.1)	0.90 (2.15)
P/S-Ferritin levels	30,106 (40.8)	1.47 (3.81)	32,094 (48.4)	1.69 (3.60)	321,087 (25.6)	0.61 (2.08)	619,138 (46.4)	1.36 (2.67)
P/S-Cobalamin (B12)	33,665 (45.7)	1.41 (2.46)	34,483 (52.0)	1.66 (2.58)	363,661 (28.9)	0.66 (1.55)	586,364 (43.9)	1.17 (2.10)
P/S-Folate	25,083 (34.0)	0.81 (1.72)	26,565 (40.0)	0.98 (1.83)	256,015 (20.4)	0.38 (1.08)	457,117 (34.2)	0.73 (1.51)
P/S-Homocysteine	10,310 (14.0)	0.23 (0.74)	11,014 (16.6)	0.27 (0.78)	94,994 (7.6)	0.11 (0.51)	165,486 (12.4)	0.19 (0.63)
B-ESR	34,530 (46.8)	1.83 (4.24)	33,828 (51.0)	2.16 (4.79)	326,110 (26.0)	0.70 (2.46)	465,591 (34.9)	1.13 (3.32)
P/S-CRP	54,527 (74.0)	14.4 (24.6)	50,297 (75.8)	13.4 (22.0)	658,660 (52.4)	3.4 (10.3)	772,872 (57.9)	3.8 (10.0)
P-Glucose	53,287 (72.3)	7.2 (12.4)	47,799 (72.1)	6.1 (10.3)	659,055 (52.4)	2.4 (5.4)	758,405 (56.8)	2.6 (5.1)
P/S-Creatinine	62,675 (85.0)	23.3 (31.4)	55,755 (84.1)	21.4 (28.1)	812,920 (64.7)	5.6 (13.3)	920,692 (68.9)	6.0 (12.6)
eGFR relative to patient	51,210 (69.5)	13.5 (22.4)	46,070 (69.5)	12.3 (20.2)	642,171 (51.1)	3.0 (8.0)	747,872 (56.0)	3.1 (7.4)
S 25-Hydroxy Vitamin D	11,377 (15.4)	0.32 (1.10)	17,770 (26.8)	0.66 (1.67)	162,259 (12.9)	0.23 (0.87)	316,576 (23.7)	0.51 (1.36)
U-Albumin/Creatinine Ratio	18,176 (24.7)	0.81 (2.33)	12,979 (19.6)	0.54 (1.91)	138,728 (11.0)	0.33 (1.52)	136,905 (10.3)	0.27 (1.35)
P/S-Urea	20,797 (28.2)	1.85 (8.74)	15,382 (23.2)	1.23 (6.56)	92,699 (7.4)	0.42 (4.70)	89,722 (6.7)	0.28 (3.57)
P/S-Uric Acid	25,402 (34.5)	1.29 (3.66)	19,165 (28.9)	0.92 (3.10)	202,899 (16.1)	0.40 (1.57)	226,301 (16.9)	0.40 (1.55)
P/S-Calcium	50,504 (68.5)	7.3 (14.1)	47,358 (71.4)	8.1 (13.5)	464,196 (36.9)	1.3 (4.6)	597,610 (44.8)	1.6 (4.4)
P/S-Ionized Calicium	15,412 (20.9)	0.62 (2.48)	18,705 (28.2)	0.94 (3.28)	90,089 (7.2)	0.14 (1.02)	158,830 (11.9)	0.25 (1.31)
P/S-TSH	46,855 (63.6)	2.9 (4.8)	48,457 (73.1)	4.9 (6.6)	601,013 (47.8)	1.5 (2.9)	871,436 (65.3)	3.3 (5.1)
U-Glucose (test strip)	18,652 (25.3)	0.87 (2.98)	16,672 (25.1)	0.95 (3.20)	147,730 (11.8)	0.33 (1.86)	198,925 (14.9)	0.45 (2.20)
U-Acetoacetate (test strip)	16,722 (22.7)	0.77 (2.87)	14,962 (22.6)	0.83 (3.05)	130,859 (10.4)	0.30 (1.80)	174,939 (13.1)	0.39 (2.09)
U-Erythrocytes (test strip)	18,659 (25.3)	0.86 (2.96)	16,693 (25.2)	0.94 (3.19)	147,480 (11.7)	0.33 (1.84)	199,285 (14.9)	0.45 (2.19)
U-Albumin (test strip)	18,857 (25.6)	0.88 (2.98)	16,830 (25.4)	0.96 (3.22)	149,538 (11.9)	0.34 (1.86)	200,809 (15.0)	0.45 (2.20)
U-Bacteria, Nitrite (test strip)	18,554 (25.2)	0.85 (2.94)	16,644 (25.1)	0.94 (3.18)	146,374 (11.6)	0.33 (1.84)	198,991 (14.9)	0.45 (2.19)
U-Leukocytes (test strip)	18,617 (25.3)	0.86 (2.95)	16,696 (25.2)	0.95 (3.20)	146,913 (11.7)	0.33 (1.84)	199,508 (14.9)	0.45 (2.19)
P/S-Sodium	59,786 (81.1)	17.6 (26.9)	53,252 (80.3)	16.6 (24.6)	692,380 (55.1)	3.8 (10.7)	798,384 (59.8)	4.1 (10.2)
P/S-Potassium	56,872 (77.2)	16.5 (26.1)	50,368 (75.9)	15.3 (23.5)	636,449 (50.6)	3.5 (10.3)	735,099 (55.0)	3.7 (9.7)
P/S-Albumin	52,697 (71.5)	10.0 (18.1)	49,086 (74.0)	10.4 (16.8)	468,921 (37.3)	1.8 (6.5)	589,794 (44.2)	2.0 (6.2)
P/S-Bilirubin	46,694 (63.3)	7.0 (14.8)	44,055 (66.4)	6.9 (13.1)	379,718 (30.2)	1.2 (5.0)	446,948 (33.5)	1.2 (4.5)
P/S-ALT	56,741 (77.0)	10.7 (16.9)	51,690 (77.9)	10.4 (15.4)	695,881 (55.4)	3.0 (7.2)	795,529 (59.6)	3.4 (7.3)
P/S-GGT	49,681 (67.4)	5.9 (11.5)	44,999 (67.8)	5.7 (10.4)	529,121 (42.1)	1.7 (4.8)	563,267 (42.2)	1.5 (4.1)
P/S-ALP	51,757 (70.2)	7.1 (12.2)	46,804 (70.6)	6.4 (10.7)	493,533 (39.3)	1.5 (4.6)	584,245 (43.8)	1.6 (4.4)
P/S-Pancreatic Amylase	32,005 (43.4)	2.12 (6.40)	28,955 (43.7)	1.89 (5.26)	266,205 (21.2)	0.60 (2.98)	320,282 (24.0)	0.66 (2.77)
P/S-LD	35,275 (47.9)	4.23 (10.16)	33,231 (50.1)	4.20 (9.56)	153,867 (12.2)	0.33 (1.99)	183,794 (13.8)	0.34 (1.90)
P/S-Protein Fractions^a^	4815 (6.5)	0.12 (0.85)	4422 (6.7)	0.11 (0.72)	28,307 (2.3)	0.03 (0.26)	42,451 (3.2)	0.04 (0.31)
U-Protein Fractions/Creatinine^a^	1547 (2.1)	0.03 (0.37)	1203 (1.8)	0.03 (0.29)	6134 (0.5)	0.01 (0.10)	7381 (0.6)	0.01 (0.11)
P/S-FLC-Kappa^b^	2638 (3.6)	0.33 (3.71)	1848 (2.8)	0.27 (3.32)	7831 (0.6)	0.01 (0.49)	8316 (0.6)	0.01 (0.47)
P/S-FLC-Lambda^b^	2497 (3.4)	0.33 (3.71)	1725 (2.6)	0.26 (3.32)	6543 (0.5)	0.01 (0.49)	6498 (0.5)	0.01 (0.47)
P/S-FLC-Ratio^b^	2497 (3.4)	0.33 (3.71)	1725 (2.6)	0.26 (3.32)	6543 (0.5)	0.01 (0.49)	6498 (0.5)	0.01 (0.47)
P/S-PSA	46,103 (62.5)	5.17 (7.37)	N/A	N/A	335,081 (26.7)	0.88 (2.31)	N/A	N/A

ALP, Alkaline Phosphatase; ALT, Alanine Aminotransferase; B, blood; CRP, C-Reactive Protein; eGFR, Estimated Glomerular Filtration Rate; ESR, Erythrocyte Sedimentation Rate; EVF, Eosinophil Volume Fraction; FLC, Free Light Chains; GGT, Gamma-Glutamyl Transferase; LD, Lactate Dehydrogenase; MCH, Mean Corpuscular Haemoglobin; MCV, Mean Corpuscular Volume; N/A, not applicable; P, plasma; PSA, Prostate-Specific Antigen; S, serum; SD, standard deviation; TSH, Thyroid Stimulating Hormone; U, urinary. ^a^ No data for Karolinska University Laboratory. ^b^ No data for SYNLAB

Among cases, 87.3% of men and 86.4% of women had B-Haemoglobin tests, with an average of approximately 24 tests per person. This is considerably higher than in controls, where 71.9% of men and 76.8% of women were tested, with an average of about 6–7 tests per person. These figures suggest that individuals classified as cases had a markedly higher frequency of B-Haemoglobin tests compared to controls, which may reflect a greater disease burden or need for closer medical follow-up in this group. However, it is also notable that a substantial proportion of controls underwent haemoglobin testing, underscoring the routine nature of this test in standard healthcare practices.

The cohort includes additional laboratory tests routinely associated with cancer investigations, as detailed in Table 5. These tests cover a broad spectrum of parameters, including complete blood cell counts, biochemical markers, vitamins, electrolytes, kidney and liver function assessments, and urinary markers, reflecting comprehensive clinical data available for analysis.

## Discussion

The STEADY-CAN cohort includes > 2.7 million individuals from the Stockholm region over a ten-year period, providing near-complete coverage of the adult population and enhancing the generalizability of findings. The cohort includes detailed data from primary and specialist care, inpatient, and outpatient visits, as well as comprehensive laboratory tests and demographic data, enabling the study of complex associations between symptoms, diagnoses, and outcomes. Comparable cohorts, such as the Clinical Practice Research Datalink (CPRD) in the United Kingdom, provide extensive primary care data with covering approximately 60 million patients, thus offering much broader national population reach [30]. However, differences in healthcare systems may impact data capture and generalizability, and CPRD lacks the specialist and detailed laboratory data available in STEADY-CAN, which can limit analyses of symptoms and early cancer detection in a clinical context.

The availability of data over a decade allows for the analysis of long-term health outcomes, crucial for studying patterns of symptoms, signs, and early cancer detection. By including the CCI and additional measures capturing both physical and mental health conditions, STEADY-CAN offers a comprehensive view of the patients’ disease burden. The Stockholm CREAtinine Measurements (SCREAM) cohort, also gathering data from Stockholm county, focuses on kidney disease and chronic illnesses but shares STEADY-CAN’s advantage of linking laboratory values to clinical outcomes [31]. With > 140,042 incident cancer cases during the ten-year outcome period, STEADY-CAN provides sufficient statistical power to identify risk patterns and early signs of cancer in the population.

Population mobility, such as individuals moving in or out of the region, may introduce selection bias [32], particularly among those with chronic conditions, which may be less prone to move. Since the cohort is based on individuals who have sought healthcare, this may lead to an overrepresentation of more severe cases, potentially affecting the generalizability of the findings to healthier populations. However, this is also the setting where a potential risk assessment tool is to be used in the future. Boennelykke’s (2022) study in Denmark, which focuses on new-onset anaemia as an indicator for cancer, also uses a healthcare-seeking sample but with a narrower focus than STEADY-CAN, which may impact generalizability for broader analyses [33].

Information bias [34], a common issue in real-world data, may arise from variable inaccuracy in diagnostic coding, impacting disease identification, especially for underdiagnosed conditions like mental health disorders. Despite comprehensive data on laboratory tests and diagnoses, information on key clinical variables such as lifestyle factors and family history are lacking. Comparable cohorts, such as CPRD, face similar challenges where the lack of lifestyle data may impact the ability to conduct risk factor analyses for chronic diseases and cancer [30]. However, some of these factors may be indirectly captured through specific comorbidities.

In contrast to UK Biobank [35], which is a research cohort collected under controlled conditions with extensive genetic and lifestyle data, STEADY-CAN provides a real-world data-based perspective on early cancer detection within a clinical care setting, enabling analyses of disease symptoms and healthcare utilization patterns that are directly applicable to practical care. This difference makes STEADY-CAN valuable for understanding the manifestation of early symptoms within a real-world healthcare setting, where diagnostics and treatments are influenced by practical clinical considerations and individual patient health context.

Temporal bias is a concern, as changes in clinical guidelines, diagnostic criteria, and healthcare practices over the study period (2011–2021) could affect data consistency [36]. Variations in cancer detection methods or updates in management guidelines may alter the frequency and approach to care, influencing the comparability of data over time and its relevance to current clinical practice.

## Conclusions

To the best of our knowledge, STEADY-CAN is one of the first large-scale population-based cohort studies to specifically investigate early cancer detection. It offers a unique opportunity to monitor and evaluate cancer detection across diverse healthcare settings, encompassing a wide range of clinical data, including diagnostic codes and laboratory results. This allows for a robust exploration of early cancer diagnosis, including incidental findings and symptom-based detection, while also facilitating adjustments for confounders. The comprehensive real-world-dataset, spanning both inpatient and outpatient care, makes STEADY-CAN a valuable tool for understanding healthcare patterns and the role of comorbid conditions in relation to early cancer detection.

## Data Availability

The data from this cohort are available upon reasonable request. Data access is governed by data sharing agreements that comply with relevant privacy and ethical guidelines. The researchers behind the cohort welcome collaboration with external researchers. Proposals for collaborative projects will be reviewed based on their scientific merit and alignment with the cohort’s objectives.

## Abbreviations

CCI: Charlson comorbidity index
GP: General Practitioners
ICD-10: International classification of diseases and related health problems, 10th revision
PHC: Primary health care
PIN: Personal identification number
SCR: Swedish cancer registry
SD: Standard deviation
STEADY-CAN: Stockholm early detection of cancer study
